# Correction to: A framework for algae modeling in regulatory risk assessment

**DOI:** 10.1093/etojnl/vgae091

**Published:** 2025-01-24

This is a correction to: Rendal, C., Witt, J., Preuss, T.G., & Ashauer, R. (2023). A framework for algae modeling in regulatory risk assessment. *Environmental Toxicology and Chemistry*, 42, 1823-1838. https://doi.org/10.1002/etc.5649

The authors of the above noted paper hereby submit the following corrections.

On page 1826, Equation 4 and the text directly after, should be replaced with the following:


$$\frac{dD\left(t\right)}{dt}=k_{dr}(C(t)-D\left(t\right))$$

In Equation 4, kdr is the damage repair rate constant (d−1), C is the concentration of toxicant (mg/l), and D is the damage level (mg/l). The term f(C) in Equation 3 is then replaced by an analogous term f(D) with $f\left(D\right)=\frac{1}{1+e^{-b\cdot \left[\ln\left(D\right)-\ln\left(EC50\right)\right]}}$.

On page 1829, the text:

The EP50 value can be seen as equivalent to the toxicity exposure ratio (TER). The TER is conventionally calculated as the regulatory acceptable concentration (RAC) divided by the PEC. In conventional risk assessment the AF is applied to the lowest endpoint to calculate the RAC.

Should be replaced with the following:

The EP50 value can be seen as equivalent to the toxicity exposure ratio (TER). The TER is conventionally calculated as the toxicity endpoint divided by the PEC. If the TER is larger than the relevant assessment factor, this indicates an acceptable risk.

These corrections do not change the discussion or conclusion of the paper.

